# SINGLE-SESSION BILATERAL REDUCED-SETTINGS PHOTODYNAMIC THERAPY FOR BILATERAL CHRONIC CENTRAL SEROUS CHORIORETINOPATHY

**DOI:** 10.1097/IAE.0000000000003807

**Published:** 2023-06-09

**Authors:** Laurenz J.B. Pauleikhoff, Roselie M.H. Diederen, Helena M.A. Feenstra, Reinier O. Schlingemann, Elon H.C. van Dijk, Camiel J.F. Boon

**Affiliations:** * Department of Ophthalmology, Amsterdam University Medical Centers, Amsterdam, The Netherlands;; † Eye Center, Medical Center – University of Freiburg, Faculty of Medicine, University of Freiburg, Freiburg, Germany;; ‡ Department of Ophthalmology, Leiden University Medical Center, Leiden, The Netherlands; and; § Department of Ophthalmology, University of Lausanne, Jules-Gonin Eye Hospital, Foundation Asile des Aveugles, Lausanne, Switzerland.

**Keywords:** chronic central serous chorioretinopathy, photodynamic therapy, bilateral cCSC, bilateral PDT

## Abstract

**Purpose:**

We performed a multicenter, retrospective study on patients with bilateral chronic central serous chorioretinopathy (cCSC) who received single-session bilateral reduced-settings photodynamic therapy (ssbPDT) and assessed anatomical (resolution of subretinal fluid [SRF]) and functional (best-corrected visual acuity [BCVA]) outcomes and safety.

**Methods:**

Patients who underwent ssbPDT between 01/01/2011 and 30/09/2022 were included. The resolution of SRF at first, second, and final follow-up was assessed on optical coherence tomography (OCT), and BCVA measurements were collected at these visits. When fovea-involving ssbPDT was performed, ellipsoid zone (EZ) and external limiting membrane (ELM) integrity was graded before and after treatment.

**Results:**

Fifty-five patients were included in this study. Sixty-two of hundred and eight eyes (56%) showed a complete resolution of SRF at the first follow-up, which increased to 73/110 (66%) at the final follow-up. The mean logMAR BCVA improved by −0.047 (*P* = 0.02) over follow-up. EZ integrity increased from 14/21 (67%) to 24/30 (80%) while ELM integrity increased from 22/30 (73%) to 29/30 (97%).

**Conclusion:**

Patients with cCSC with bilateral SRF at baseline showed significant anatomical and functional improvements after ssbPDT, both at short-term and long-term follow-up. No relevant adverse events were noted.

## Abstract 2

Supplemental Digital Content is Available in the Text.

Patients with bilateral chronic central serous chorioretinopathy (cCSC) who were treated with single-session bilateral reduced-settings photodynamic therapy (ssbPDT) showed good anatomical and functional outcomes with no significant adverse events. This shows that ssbPDT is a safe and effective treatment option for patients with cCSC with bilateral subretinal fluid.

Central serous chorioretinopathy (CSC) is one of the most common causes of fluid leakage in the macula^1^ and primarily affects men between the ages of 20 to 60 years. It is characterized by a central accumulation of subretinal fluid (SRF), which can cause irreversible damage to photoreceptor cells and the retinal pigment epithelium (RPE) when persisting over a longer period of time.^2^ Although often unilateral, between 25% and 50% of patients with CSC are affected bilaterally,^3^ and up to 19% of initially unilateral CSC cases develop SRF in the fellow eye later on.^4^ Subclinical abnormalities of the RPE and choroid are seen in up to 61% of fellow eyes.^5^

A key factor for the occurrence of RPE abnormalities and SRF seems to be an abnormal, dysfunctional, and thickened choroid (‘pachychoroid’). These typical choroidal changes in CSC and other diseases that are part of the pachychoroid disease spectrum show an increased choroidal thickness and enlarged Haller layer vessels on optical coherence tomography (OCT) (‘pachyvessels’),^5^ as well as prominent choroidal vessels and midphase hyperfluorescence on indocyanine green angiography (ICGA).^6^ These choroidal abnormalities may be because of an overload of the choroidal venous system,^7^ potentially due to a vortex vein drainage problem,^8^ while arteriovenous anastomoses in the posterior pole have also been hypothesized to play a role.^9^ However, it is currently unclear how these factors are linked to some of the key risk factors in CSC, most notably the male sex and corticosteroid use. Evidence from other conditions, such as dural arteriovenous fistula, shows similar associations, hinting at a possible pathogenic link.^9^

Because these choroidal abnormalities presumably lie at the basis of CSC, photodynamic therapy (PDT) is a promising treatment to combat the causal changes (choroidal congestion and hyperpermeability) and their consequences (RPE abnormalities).^6,10,11^ Recently, the prospective, randomized controlled PLACE and SPECTRA trials have shown that reduced-settings PDT achieves a significantly higher success rate for complete SRF resolution and improved functional outcomes in chronic CSC (cCSC) than other proposed therapy options, such as oral eplerenone or micropulse laser treatment.^2,12–14^ During PDT, the photosensitizer verteporfin is applied intravenously and then activated after 15 minutes by a 689-nm laser in a predefined area of the retina. In many patients, this results in a remodeling of the choroid, decreasing abnormal choroidal thickness and hyperpermeability,^15^ which often reduces the extent of RPE abnormalities,^11^ with subsequent resolution of SRF.^2^

However, a global shortage of verteporfin has recently led to reduced access to PDT and a worldwide delay of treatment.^16^ Moreover, verteporfin is a relatively expensive drug, and because it is used as an off-label treatment in cCSC, reimbursement of the treatment can be an issue in some countries. It is, therefore, essential to use the available verteporfin as efficiently as possible. This can be achieved by treating both eyes in bilateral cCSC during a single treatment session, for which only one dose of verteporfin has to be injected and is then activated sequentially using the laser in both eyes. However, to the best of our knowledge, no large case series on single-session bilateral reduced-settings photodynamic therapy (ssbPDT) being performed in patients suffering from cCSC have been published yet.

The goal of this study was, therefore, to describe patients with bilateral cCSC who received ssbPDT and to analyze both the anatomical and functional outcomes as well as the safety of the intervention.

## Methods

### Patients

A multicenter, retrospective study was performed on patients with clinically confirmed cCSC, who received at least one ssbPDT at the tertiary referral centers Amsterdam University Medical Centers (AUMC) and Leiden University Medical Center (LUMC) and for whom at least one follow-up visit was available.

### Clinical Examinations

Electronic medical records of the patients were screened for patient demographics, ophthalmic medical history, best-corrected visual acuity (BCVA), and PDT settings including the eye that first received PDT. The diagnosis of cCSC was made based on the definitions of the PLACE, SPECTRA, and VICI trials, which included SRF visible on an OCT scan, one or more regions of active focal leakage combined with RPE window defects visible on fundus fluorescein angiography (FFA), and hyperfluorescent changes on ICGA.^12–14^ To assess the severity of cCSC, pretreatment presence of diffuse atrophy of the retinal pigment epithelium (DARA), defined as large window defects on midphase FFA, and posterior cystoid retinal degeneration (PCRD), defined as cystoid cavities on OCT without associated leakage on FFA, were assessed. Imaging examples for each of these phenomena have been previously published.^17^ The presence of pachyvessels (defined as large Haller layer vessels) and of subfoveal SRF were also assessed based on OCT, which was performed using either Heidelberg SPECTRALIS or HRA2 (both Heidelberg Engineering, Heidelberg, Germany), Topcon 3D OCT-2000 (Topcon, Tokyo, Japan) or Stratus OCT 3000 (Carl Zeiss AG, Jena, Germany). FFA patterns were graded as either showing focal or diffuse leakage.^18,19^

### PDT Treatment

Patients underwent half-dose ssbPDT (3 mg/m^2^ verteporfin [Visudyne, CHEPLAPHARM Arzneimittel GmbH, Greifswald, Germany]) compared with the original PDT settings described for neovascular age-related macular degeneration (nAMD). The treatment followed a standardized protocol similar to the treatment protocols of the PDT arm in the SPECTRA and PLACE trials.^12,13^ In brief, the area of treatment was defined by the respective surgeon based on the extent of choroidal hyperpermeability as seen on midphase ICGA.^12,13^ After instillation of anesthetic eye drops, a contact lens (×1.5 magnification) (Volk Optical) was positioned on one eye and the verteporfin was activated with a 689-nm laser (Vitra 689, Quantel Medical, Cournon d’Auvergne, France). The treating physician decided which eye was treated first. The fellow eye was treated immediately afterward, resulting in a slight delay of treatment due to the positioning of the lens and the refocus of the treatment spot. Patients were advised to protect their skin and eyes by wearing protective skin-covering clothing and sunglasses for the first 48 hours after half-dose PDT was performed, as is standard practice for unilateral PDT.

In case a patient underwent multiple ssbPDT treatments, the first session with complete electronic patient records was selected.

### Clinical Outcome Measures

The primary outcome measure was the amount of residual SRF after ssbPDT. It was graded as no response (defined as no detectable decrease in SRF after treatment), incomplete resolution, or complete resolution (defined as no more discernible subretinal fluid) at first, second, and final follow-up. Secondary outcome measures included change in logarithm of the minimum angle of resolution (logMAR) BCVA and treatment response for the eye treated first compared with that treated second within the single-session treatment sequence. Moreover, the impact of factors such as history of PDT, previous other CSC treatment, previous anti-VEGF injections, presence of PCRD, FFA leakage pattern, order of treatment, presence of pachyvessels, and presence of SRF on treatment response were analyzed in a subgroup analysis.

### Safety of PDT Treatment

Moreover, the safety of the procedure was assessed by investigating patients with 1-line or more drop in BCVA at the first follow-up and those with 3-lines or more drop at the final follow-up. In these patients, patient records were screened for comorbidities and subjective changes in visual acuity.

To exclude the development of atrophy in the area treated with ssbPDT, the integrity of the external limiting membrane (ELM) and ellipsoid zone (EZ) was graded as described before.^20^ In brief, eyes that had undergone fovea-involving ssbPDT and showed complete SRF resolution at the first follow-up and no recurrence, ELM and EZ integrity was graded as continuous/regular, interrupted/irregular, or indiscernible before and after treatment. Examples of these definitions were previously published.^20^ When fovea-involving SRF was present at the preoperative visit, only ELM integrity was assessed.

### Statistical Analysis

Statistical analysis was performed using R software.^21^ BCVA measured in Snellen charts was converted to logMAR BCVA using LogMAR = −log (Snellen fraction). BCVA readings below the Snellen scale (counting fingers) were converted to their approximate logMAR values using an Excel spreadsheet previously published.^22^ Statistical significance was tested using a paired *t*-test for the change in logMAR BCVA and Pearson chi-square test for treatment responses and subgroup analyses. A *P* value of < 0.05 was considered statistically significant for change in BCVA, and an adjusted *P* value of < 0.002 after Bonferroni correction was considered significant for the subgroup analyses.

## Results

### Patient Demographics

Fifty-five patients who received ssbPDT for bilateral cCSC between 01/01/2011 and 30/09/2022 were included in this study. Their baseline characteristics are summarized in Table 1. The mean age at presentation was 51.1 (±12.4) years. Fourty-seven patients were male (85%) and eight were female (15%). The median duration of symptoms was 8 months and varied significantly (IQR 4–48 months). The median initial BCVA was similar in both eyes (0.13 LogMAR OD and 0.05 LogMAR OS, corresponding to between 20/20 and 20/30 Snellen VA). However, seven eyes of seven patients (13%) showed a BCVA of 20/200 or less. In two eyes (4%), this was attributed to amblyopia, while the other five eyes (9%) showed extensive PCRD and photoreceptor damage.

**Table 1.** Demographic Characteristics of the Patients with Chronic Central Serous Chorioretinopathy Included in This Study

Patient Demographics	
Age (yrs)	51.1 ± 12.4
Sex	
Male	47/55 (85%)
Female	8/55 (15%)
Duration of symptoms (months)	8 (4–48)
Previous use of steroids	
Systemic	7/43 (16%)
Topical	8/43 (19%)
None	28/43 (65%)
Initial BCVA LogMAR	
OD	0.13 (0–0.28)
OS	0.05 (0–0.30)
Diffuse RPE atrophy in at least 1 eye	
Yes	33/50 (66%)
No	17/50 (34%)
Posterior cystoid retinal degeneration in at least 1 eye	
Yes	16/55 (29%)
No	39/55 (71%)
FFA leakage pattern	
OD	
Focal	35/50 (70%)
Diffuse	15/50 (30%)
OS	
Focal	30/50 (60%)
Diffuse	20/50 (40%)
Presence of pachyvessels	
OD	
Yes	29/41 (71%)
No	12/41 (29%)
OS	
Yes	27/40 (68%)
No	13/40 (32%)

The mean age varied significantly within the cohort. Preoperative FFA imaging was available for 50 patients, and data on previous usage of corticosteroids were documented in 43 patients. Age is presented as mean ± SD while duration of symptoms and logMAR BCVA are presented by median (interquartile range). The presence of pachyvessels in Haller layer was assessed on en face OCT.

BCVA, best-corrected visual acuity; LogMAR, logarithm of the minimum angle of resolution; RPE, retinal pigment epithelium; FFA, fundus fluorescein angiography; OD, right eye; OS, left eye.

Regarding imaging characteristics, 16 of 55 patients (29%) showed PCRD in at least one eye, of whom five (5 of 16, 31%) showed bilateral PCRD. Most of the patients (33 of 50, 66%) showed DARA in at least one eye, with 76% (25 of 33) of those exhibiting bilateral DARA. The leakage patterns showed diffuse leakage in 35 of 100 eyes (35%) while 65 eyes (65%) showed focal leakage with a median of 2 leakage points (range 1–7).

Previous other CSC treatments included previous PDT (17 of 55, 31%) or an alternative laser treatment (conventional or micropulse laser, 9 of 55, 16%). Three patients (5%) had received eplerenone before PDT while two patients (4%) had been treated with prednisolone eye drops for PCRD.

Sixteen eyes of twelve patients had received intravitreal antivascular endothelial growth factor injections because of a suspicion of choroidal neovascularization (CNV) in five eyes (31%) of four patients based on multimodal imaging while in three eyes (19%) of two patients, the referring ophthalmologist had initiated these injections, but no CNV was detected by the tertiary referral center. In eight eyes (50%) of six patients, injections were performed as a treatment attempt for PCRD.

### Treatment Response

The first follow-up was approximately 10 weeks after PDT (median 69 days, IQR 57–77) and was completed by all but one patient while the second follow-up was approximately 5 months after PDT (median 157 days, IQR 139–210), but was missed by 13 patients. Overall median follow-up was more than two years (median 755 days, IQR 251–1196), but up to 11 years in individual patients. PDT procedures were performed by five surgeons. The mean PDT spot size was 5.2 ± 1.5 mm.

**Fig. 1.**
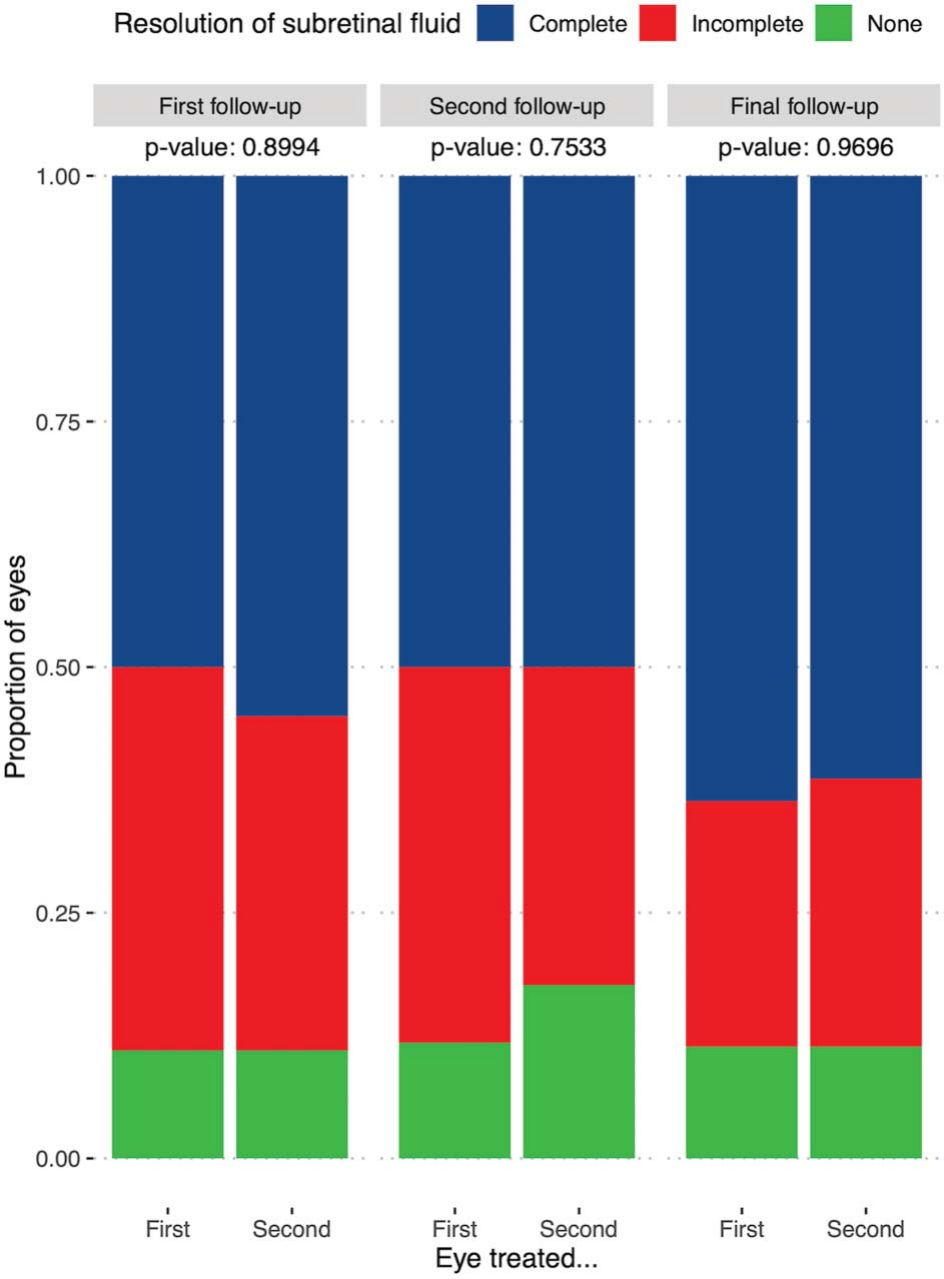
Treatment response of chronic central serous chorioretinopathy eyes treated first or second during single-session bilateral reduced-settings photodynamic therapy. The proportion of eyes with complete (blue) or incomplete (red) resolution of subretinal fluid or no treatment response (green) on the first (left), second (middle), or final (right) follow-up visits. No clear difference in treatment response between eyes treated first or second was observed.

On the first follow-up, 62 of 108 eyes (56%) showed complete SRF resolution, which dropped slightly to 42% for the second follow-up and increased again to 66% (73/110) at the final follow-up. Accordingly, the share of eyes with incomplete SRF resolution dropped from 32% at the first follow-up to 24% at the final follow-up. Approximately 10% of eyes showed no significant change in SRF. For the 44 patients for whom it was recorded, the order of treatment did not have a striking effect on treatment response (see Figure 1). Neither a previous PDT, previous other CSC treatments, FFA leakage pattern, the presence of PCRD, the order of treatment, the presence of pachyvessels, nor subfoveal SRF had a statistically significant effect on treatment response after adjusting for multiple testing (see Table 2). A history of anti-VEGF injections and thus suspected secondary CNV resulted in a higher percentage of eyes showing no response (31% vs. 6%), which was, however, also not statistically significant.

**Table 2.** Analysis of Different Factors and Their Impact on Treatment Response

Patient Characteristic	Subretinal Fluid Treatment Response											
	First Follow-up			*P*	Second Follow-up			*P*	Final Follow-up			*P*
	Complete	Incomplete	No Response		Complete	Incomplete	No Response		Complete	Incomplete	No Response	
Previous PDT												
Yes	59%	23%	18%	0.280	45%	45%	10%	0.291	61%	22%	17%	0.414
No	57%	35%	8%		58%	27%	16%		68%	24%	8%	
Previous other CSC treatments												
Yes	54%	15%	31%	0.023	23%	62%	15%	0.024	62%	15%	23%	0.223
No	58%	35%	7%		61%	25%	14%		67%	25%	8%	
Previous anti-VEGF injections												
Yes	50%	31%	19%	0.463	50%	31%	19%	0.836	50%	19%	31%	0.009
No	59%	33%	9%		56%	31%	13%		69%	25%	6%	
Presence of PCRD												
Yes	55%	25%	20%	0.251	44%	28%	28%	0.179	52%	29%	19%	0.206
No	58%	34%	8%		58%	32%	11%		70%	22%	8%	
Leakage pattern												
Focal	55%	37%	8%	0.305	55%	30%	15%	0.892	55%	37%	8%	0.305
Diffuse	61%	24%	15%		59%	30%	11%		61%	24%	15%	
Order of treatment												
First	50%	39%	11%	0.899	50%	38%	12%	0.753	64%	25%	11%	0.970
Second	55%	34%	11%		50%	32%	18%		61%	27%	11%	
Presence of pachyvessels*												
Yes	54%	32%	14%	0.142	47%	33%	21%	0.155	66%	23%	11%	0.485
No	57%	43%	0%		69%	31%	0%		56%	36%	8%	
Subfoveal fluid present												
Yes	53%	38%	9%	0.282	57%	30%	13%	0.891	59%	31%	10%	0.046
No	64%	23%	13%		52%	32%	16%		79%	11%	11%	

The treatment response is shown as percentage of eyes with complete or incomplete subretinal fluid resolution or no response according to different subgroups. These were the eyes treated first or second, those that received previous anti-VEGF injections, those that showed a focal or diffuse leakage pattern, those that had undergone previous PDT, or those that had other previous CSC treatments, such as eplerenone, focal laser, or micropulse laser. All *P* values were calculated using the Pearson chi-square test. Adjusted *P* values (Bonferroni correction) of < 0.002 were considered statistically significant.

* Pachyvessels were only gradable in 81 of 110 eyes because of image quality.

PDT, photodynamic therapy, CSC, central serous chorioretinopathy, VEGF, vascular endothelial growth factor.

Regarding the treatment effect of PDT on PCRD itself, three eyes (15%) of three patients showed a complete resolution of PCRD at the first follow-up, 11 eyes (55%) of eight patients showed an incomplete resolution, and six eyes (30%) of six patients showed no treatment response.

The logMAR BCVA improved by −0.026 (*P* = 0.08) at the first follow-up and remained at that level at the second follow-up (−0.022 (*P* = 0.21)). At the final follow-up, a statistically significant improvement of −0.047 (*P* = 0.02) was observed.

Most of the eyes (69%, 76 of 110) did not require any further treatment during follow-up because of the absence of SRF. However, 25% of eyes (27 of 110) underwent one additional PDT because of incomplete SRF resolution (33%, 9/27) or SRF recurrence (67%, 18/27). Seven eyes (7 of 110, 6%) of six patients received two or more additional PDTs, which resulted in complete SRF resolution in 71% of eyes (5/7) at the final visit.

### Safety

Nine eyes (9 of 108, 8%) of eight patients showed a 1-line drop in BCVA at the first follow-up. Of these, three eyes of three patients showed spontaneous recovery by the second follow-up. Two eyes of two patients showed a decrease in BCVA from −0.20 (20/12.5) to −0.09 (20/16) without any subjective change in visual acuity. The remaining four eyes of three patients all had either significant comorbidities (amblyopia, traumatic eye injury) or extensive PCRD and showed a 1-line drop in BCVA from a low initial BCVA (between 20/50 and 20/200) without a subjective decline in vision.

Two eyes (2%) of two patients showed a 3-line or more drop in BCVA during follow-up, both of which presented with an initial BCVA of 1.0 (20/200) in the regarding eye and showed extensive PCRD at the initial presentation. Treatment was primarily focused on the fellow eye, and patients were informed on the guarded prognosis of treating the second eye. In one case, BCVA dropped to 1.3 (20/400) at the first and second follow-ups and finally to counting fingers 8 months after ssbPDT. In the second case, the patient was not seen for the first and second follow-ups, but was re-referred 7 years after the initial treatment was performed, when his BCVA had dropped to 2/60.

EZ and ELM integrity was assessed before treatment and at the final follow-up in 30 eyes (27%) with fovea-involving ssbPDT. The proportion of eyes with a continuous ELM increased from 73% pre-PDT to 97% while that with continuous EZ increased from 67% to 80% (see Table, Supplemental Digital Content 2, http://links.lww.com/IAE/B970). A posttreatment occurrence or increase of a foveal hyperreflective “comet tail” sign of increased OCT transmission due to RPE atrophy was not observed.

## Discussion

In this multicenter study of patients with bilateral cCSC who received ssbPDT, we were able to show that it is a safe and effective treatment option.

The use of ssbPDT was not part of the large trials on PDT for nAMD,^23^ or secondary choroidal neovascularization in pathological myopia,^24^ and has thus only been studied in smaller case studies or series. Some reports have studied its use in cases of bilateral CNV secondary to Stargardt disease,^25^ optic nerve drusen,^26^ or macular telangiectasia,^27^ as well as for idiopathic pigment epithelial detachment,^28^ all of which reported favorable anatomical and functional outcomes, be it for a small number of patients. However, to the best of our knowledge, the use of ssbPDT in cCSC has not been reported so far. Moreover, these reports performed full-dose and full-fluence PDT. By contrast, in CSC, it was shown that a reduced-settings PDT (either half-dose, half-fluence, or half-time compared with the original protocol) results in similar functional outcomes, but fewer deleterious effects, such as choriocapillaris hypoperfusion and retinal thinning.^29^ Reduced-settings PDT is, therefore, preferred for the treatment of CSC cases^2^ and was performed in all patients in this study.

One possible concern regarding ssbPDT is the delay in treatment between the eye treated first and the one treated second. Because the laser treatment is supposed to start at exactly 15 minutes after the commencement of the verteporfin injection and each spot is applied for 83 seconds, there is an inevitable small time lag between both eyes, which could result in a reduced treatment efficacy in the second eye. The largest previous study on this issue investigated 20 patients with bilateral nAMD who received ssbPDT and compared outcomes between eyes treated first versus eyes treated second.^30^ It noted a good treatment response in both eyes with similar efficacy. However, the results were limited by the small sample size, besides studying a disease that is very different from cCSC. Our study in 55 patients with cCSC confirms those results indicating that the small time lag is of limited importance.

Regarding bilateral cCSC in general, little is known about its differences compared with unilateral cCSC. Spaide et al^3^ found that patients with CSC older than 50 years were more likely to have a bilateral presentation compared with younger patients. Of note, they also reported that DARA was more common in older patients with CSC, which may explain the high percentage of patients affected by DARA (66%) in our cohort. Whether the visual prognosis of bilateral cCSC differs from unilateral cases, however, has thus far not been studied. Based on our study, the median final logMAR BCVA of 0 (= 20/20 Snellen) (IQR −0.08 to 0.22) highlights that after adequate treatment, a good BCVA can be achieved in most patients with bilateral cCSC.

Interestingly, patients in our cohort share many similarities with a cohort of “severe” cCSC cases that we previously reported.^17^ Both studies included many patients with PCRD and DARA, which are commonly considered hallmarks of advanced cCSC.^31,32^ Regardless of the severity, however, both studies showed a significant increase in BCVA after PDT and complete SRF resolution in the most of the patients. This study also highlights that even during a long follow-up of up to 11 years, patients still reported an overall improvement in BCVA and favorable anatomical results. Even “severe” phenotypes of cCSC can thus safely be treated using PDT.

Comparing treatment outcomes in this study with those in the PLACE trial, a similar percentage of eyes showed complete SRF resolution at the first follow-up (56% vs. 51%), but a less pronounced effect was seen at the second follow-up (42% vs. 67%).^12^ At the final follow-up, 66% of eyes in this study showed complete SRF resolution, which is still lower than that in several other large studies (up to 78% success rate).^2,13^ Reasons for this difference in treatment response could be the severely affected cCSC eyes with a BCVA below 20/200 included in this study (7 eyes), which could not have been included in those trials, or a more severe phenotype of bilateral cCSC compared with unilateral disease in general.

Our study also focused on the safety of ssbPDT. We did not observe a significant deterioration in BCVA in eyes that had a good initial BCVA. Whether the deterioration observed in a few eyes with a lower initial BCVA can be attributed to their comorbidities (e.g., previous ocular trauma or amblyopia), the natural course of the disease or the treatment is not entirely certain. We did, however, not observe new RPE atrophy in any of the patients where OCT grading was performed. Moreover, more patients showed ELM and EZ integrity after ssbPDT than before treatment.

Interestingly, some patients in this study received one or more additional PDTs. To date, the efficacy and, especially, safety of multiple PDTs have not been studied in cCSC. Some small studies reported multiple PDTs for CNVs (e.g., 3 times with full-dose settings as opposed to the reduced-settings used in our study)^27,30^ and did not report the development of RPE atrophy, but instead still noted favorable functional outcomes in most patients. Patients treated with multiple PDTs in this study often had significant FAF abnormalities before the first of those treatments, but did not show a progression of atrophy after treatment (see Figure, Supplemental Digital Content 1, http://links.lww.com/IAE/B969). This is further supported by evidence from our group that patients included in the PLACE trial did not develop RPE atrophy at 2 years after PDT.^20^

Of note, 16 eyes of 12 patients had received intravitreal anti-VEGF injections. Only in a small minority of these, however, a secondary CNV was confirmed by the tertiary referral center (five eyes of four patients). In most patients, the injections were initiated because of a presence of PCRD, highlighting a more severe CSC phenotype in these patients. Nevertheless, we saw no statistically significant difference in anatomical PDT response between patients who received anti-VEGF injections and those who did not. At the final visit, however, a lower numerical percentage of patients who had received anti-VEGF injections showed complete SRF resolution (50% vs. 69%). Whether this was due to CNV activity or a more severe phenotype in this group remains to be elucidated. Of note, a good PDT response in patients with CNV secondary to CSC was previously reported,^33^ with functional outcomes comparable with anti-VEGF treatment,^34^ but a greater reduction in central macular thickness. Large prospective randomized trials that compare PDT and intravitreal anti-VEGF injections for secondary CNV in CSC, therefore, seem warranted.

Limitations of this study include its retrospective nature, which encompasses incomplete multimodal imaging or limited follow-up for some patients, although we were able to include a relatively large number of patients and a follow-up of up to 11 years.

In conclusion, we show that ssbPDT is an effective and safe treatment option in patients with bilateral cCSC. This can be of real-life benefit to both patients and doctors by decreasing treatment costs, reducing the overall number of PDTs needed in bilateral cCSC, and optimizing verteporfin use in times of a global shortage.

## Supplementary Material
