# Supplementary figures and images for: SINGLE-SESSION BILATERAL REDUCED-SETTINGS PHOTODYNAMIC THERAPY FOR BILATERAL CHRONIC CENTRAL SEROUS CHORIORETINOPATHY
Source: Retina. 2023 Jun 9;43(8):1356–63. doi: 10.1097/IAE.0000000000003807 (PMC10627544; doi:10.1097/IAE.0000000000003807)

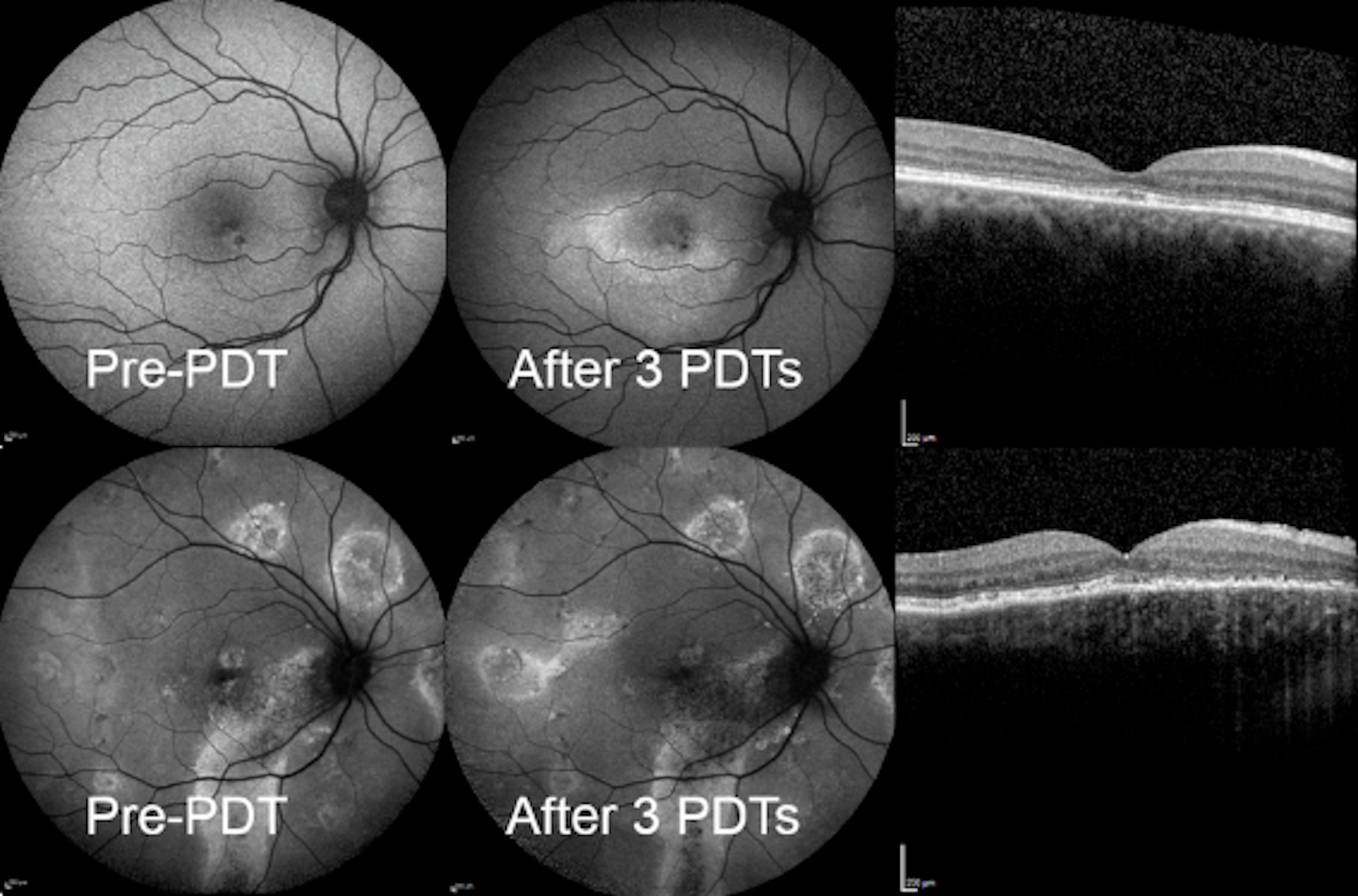

Supplement: Supplementary file 1 [file retina-43-1356-s001.tif]
