# Supplementary material for: SINGLE-SESSION BILATERAL REDUCED-SETTINGS PHOTODYNAMIC THERAPY FOR BILATERAL CHRONIC CENTRAL SEROUS CHORIORETINOPATHY
Source: Retina. 2023 Jun 9;43(8):1356–63. doi: 10.1097/IAE.0000000000003807 (PMC10627544; doi:10.1097/IAE.0000000000003807)
Supplement: Supplementary file 2 [file retina-43-1356-s002.docx]

|  | **ELM** | | **EZ** | |
| --- | --- | --- | --- | --- |
|  | **preoperative** | **final follow up** | **preoperative** | **final follow up** |
| Continuous | 73% (22/30) | 97% (29/30) | 67% (14/21) | 80% (24/30) |
| Interrupted | 27% (8/30) | 3% (1/30) | 33% (7/21) | 17% (5/30) |
| Indiscernible | 0% (0/30) | 0% (0/30) | 0% (0/21) | 3% (1/30) |

**Supplementary table 1: Grading of foveal external limiting membrane (ELM) and ellipsoid zone (EZ) integrity in eyes with fovea-involving PDT.** Both ELM and EZ were graded as either continuous without significant abnormalities, interrupted / irregular when the layer was present, but did not show a regular pattern, or indiscernible in cases where the respective layer was not identifiable at all. When center-involving subretinal fluid was present at the pre-treatment visit, the EZ was not graded, since it cannot be clearly delineated.
